# Prospective observational study of the frequency and features of intra-abdominal abscesses in patients with melioidosis in northeast Thailand

**DOI:** 10.1016/j.trstmh.2012.05.007

**Published:** 2012-10

**Authors:** Rapeephan R. Maude, Teerapon Vatcharapreechasakul, Pitchayanant Ariyaprasert, Richard J. Maude, Maliwan Hongsuwan, Prayoon Yuentrakul, Direk Limmathurotsakul, Gavin C.K.W. Koh, Wipada Chaowagul, Nicholas P.J. Day, Sharon J. Peacock

**Affiliations:** a Mahidol-Oxford Tropical Medicine Research Unit, Faculty of Tropical Medicine, 3/F 60th Anniversary Chalermprakiat Bldg, 420/6 Rajvithi Road, Rajthevi, Bangkok 10400, Thailand; b Sappasithiprasong Hospital, Ubon Ratchathani, Thailand; c Centre for Tropical Medicine, Centre for Clinical Vaccinology and Tropical Medicine, Nuffield Department of Clinical Medicine, University of Oxford, Churchill Hospital, Oxford, UK; d Department of Medicine, University of Cambridge, Addenbrooke's Hospital, Cambridge, UK; e Department of Microbiology and Immunology, Faculty of Tropical Medicine, Mahidol University, Bangkok, Thailand

**Keywords:** Melioidosis, Abscess, Liver, Spleen, *Burkholderia pseudomallei*, Ultrasound

## Abstract

Retrospective case series from Thailand have reported the presence of intra-abdominal abscesses in around half of patients with melioidosis, a much higher rate than our clinical experience would suggest. We performed a prospective, observational study of 230 adult patients with culture-confirmed melioidosis in which all patients underwent abdominal ultrasound. One or more abscesses were detected in the liver and/or spleen in 77 (33%) cases. These were often multiple (70%, 31/44 in hepatic abscesses and 88%, 50/57 in splenic abscesses) and clinically silent (27% of cases with abscesses presenting with abdominal pain). The mortality rate at 4 weeks post-discharge was lower in patients who were abscess-positive vs abscess-negative (10%, 8/77 vs 20%, 31/153).

## 1 Introduction

Melioidosis is an infectious disease caused by *Burkholderia pseudomallei*, a Gram-negative bacillus present in the environment across much of Southeast Asia and in northern Australia. Infection occurs following bacterial inoculation, inhalation or ingestion and predominantly affects agricultural workers with risk factors such as diabetes mellitus and renal impairment. Retrospective case series from Thailand have reported high rates of intra-abdominal abscesses in patients with melioidosis, with around half of cases having one or more abscesses in the liver and/or spleen.^1^ This rate is much higher than our clinical experience from treating patients with melioidosis in northeast Thailand would suggest. Furthermore, we have reported lower rates in the context of a comparative drug trial in which 23% (48/212) of cases with culture-proven melioidosis had liver and/or splenic abscesses,^2^ although ultrasound was not performed in all cases. We hypothesized that the rate of intra-abdominal abscesses was lower in our setting than that reported in retrospective case series, and performed a prospective observational study to test this on the basis that an accurate estimate of frequency would contribute to our bedside understanding of the disease.

## 2 Methods

Patients with culture-confirmed melioidosis were recruited from the adult wards (aged ≥16 years) of Sappasithiprasong Hospital in Ubon Ratchathani, northeast Thailand, between 16 August 2008 and 17 August 2009. The hospital diagnostic laboratory was contacted daily to identify patients with one or more cultures positive for *B. pseudomallei*. Active surveillance for suspected cases was also performed through daily rounds of the medical and intensive care wards. Any patient suspected of having melioidosis based on presenting clinical features had samples taken for culture, including blood, respiratory secretions (sputum or tracheal aspirate if intubated), urine, throat swab, pus and surface swabs from skin lesions. Patients with microbiologically confirmed melioidosis were recruited into the study following written informed consent from the patient or next of kin. A history was taken and examination performed during the first visit, and the patient seen daily until discharge or death. An abdominal ultrasound was performed by an experienced operator on the day of recruitment, or as soon as possible thereafter. Patient outcome (survival/death) was determined 4 weeks post-discharge by telephone call and/or home visit. A minority of relatives took moribund patients home to die and these cases were followed up to confirm the outcome.

Ethical approval for this study was obtained from Sappasitthiprasong Hospital Ethics Committee. Statistical analysis was performed using STATA version 11.0 (Stata Corporation, College Station, TX, USA). Fisher exact or χ^2^ tests were used to assess categorical variables. The Student t test or Mann-Whitney U test was used for continuous variables.

## 3 Results and Discussion

There were a total of 314 adult patients admitted during the study period with suspected or culture-positive melioidosis, of whom 230 (73%) were recruited to this study. The first scan was undertaken within 48 or 72 hours of recruitment in 72% (166/314) and 86% (198/314) of cases, respectively. One or more abscesses were identified in the liver and/or spleen in 77/230 (33%) cases, 5 of who also had ultrasound evidence of abscesses at other sites (kidney, 2; prostate, 1; pancreas, 1; adrenal gland, 1). One or more abscesses were present in the liver alone in 20/77 (26%), in the spleen alone in 33/77 (43%), and in both organs in 24/77 (31%) cases. Abscesses were noted to be multiple in 31/44 (70%) and 50/57 (88%) of cases with liver and splenic abscesses, respectively. No patient developed an abscess that became clinically apparent after an initial negative scan during this study. The higher frequency of splenic abscesses and the presence of multiple abscesses are consistent with previous reports.^3^ This rate of abscess detection by ultrasound is considerably lower than the rates determined during retrospective studies in Thailand using the same imaging technology, but is more closely consistent with our clinical experience. Our findings are higher than the reported rate of intra-abdominal abscess (including splenic, liver, kidney and adrenal) in Australia of 12%.^4^ However, the incidence of prostatic abscess in this study was lower than previously reported in northern Australia.^4^ Of the 230 patients, 69 (30%) were culture-positive from blood, of whom 26 (38%) had an intra-abdominal abscess. There was no relationship between the presence of bacteraemia and the occurrence of intra-abdominal abscess (p = 0.29).

Characteristics of patients with or without abscesses on ultrasound scan are compared in Table 1. Patients with one or more abscesses on ultrasound were younger, had a higher rate of known renal disease, and were more likely to have abdominal pain and a palpable liver and/or spleen compared with patients who had a negative ultrasound scan. Abscesses were cryptic, however, in around three-quarters of cases. This provides support for the use of ultrasound scanning (or other imaging) of all patients with melioidosis.

**Table 1:** Characteristics of patients with and without abscesses

Characteristic	No abscess (n = 153)		≥1 abscesses (n = 77)		p value
Male	99	(65%)	46	(60%)	NS
Age, median (IQR)	54	(43–61)	48	(39–54)	0.02
Rice Farmer	133	(87%)	66	(86%)	NS
Diabetes	76	(50%)	48	(62%)	NS
Haematologic disease	6	(4%)	6	(8%)	NS
Chronic renal disease	1	(1%)	11	(14%)	<0.001
Chronic liver disease	6	(4%)	1	(1%)	NS
Excess alcohol consumption	11	(7%)	0	(0%)	0.02
Chronic lung disease	1	(1%)	1	(1%)	NS
Previous melioidosis	12	(8%)	8	(10%)	NS
Immunosuppressant	24	(16%)	9	(12%)	NS
Abdominal pain	22	(14%)	21	(27%)	<0.001
Palpable liver	16	(10%)	24	(31%)	<0.001
Palpable spleen	2	(1%)	7	(9%)	NS

NS: Not significant.

Incision and drainage was performed in 16 cases with large solitary abscesses. Splenectomy was performed in two cases with multiple splenic abscesses not amenable to surgical or radiologically-guided drainage. Length of stay was comparable between the patients with and without intra-abdominal abscesses (9 days [IQR 4–15 days] vs 10 days [IQR 5–19 days], p = 0.93). The mortality rate at 4 weeks post-discharge was lower in patients who were abscess positive than in patients without intra-abdominal abscesses detected (8/77 [10%] vs 31/153 [20%]), although this did not reach statistical significance (p = 0.06). This trend towards a lower mortality in patients with intra-abdominal abscesses is consistent with previous studies, and may reflect the ability of the host immune defences to localize and control infection.

An important limitation both here and in previous studies is that patients with severe disease who died within the first 24–48 hours were under-represented. Around half of all deaths from melioidosis occur within the first 48 hours, and such cases are often diagnosed retrospectively once the culture results become available. This is reflected in the overall mortality rate for the 230 study patients of 17%, which is less than half that reported from the same hospital when all cases are taken into account.^5^ Computerised tomography is more sensitive for detecting intra-abdominal abscesses than ultrasound and is used elsewhere to investigate patients with melioidosis, but our choice of ultrasound is based on the fact that many settings in Asia where melioidosis occurs may have access to ultrasound but not to computerised tomography.

In conclusion, hepatic and splenic abscesses in patients with melioidosis were often multiple and clinically silent, but mortality in patients with hepatosplenic abscesses 4 weeks post-discharge was lower than in patients without abscesses.

## Authors’ contributions

RRM, TV, PA, MH and GCKWK conceived the study. RRM, TV, PA, MH, PY, DL, GCKWK, WC and SJP designed the study. RRM and RJM analysed the data. RRJ, RJM, DL and NPJD interpreted the data. RRM, RJM and SJP drafted the manuscript. All authors critically revised the manuscript for intellectual content, read and approved the final version. SJP is the guarantor of the paper.

## Funding

This study was funded by the Wellcome Trust, London, UK (Grant number 087460/Z/08/Z).

## Competing interests

None.

## Ethical approval

Ethical approval for this study was obtained from Sappasitthiprasong Hospital Ethical Committee (Reference number 03/2008).
